# MiR-200c reprograms fibroblasts to recapitulate the phenotype of CAFs in breast cancer progression

**DOI:** 10.15698/cst2024.03.293

**Published:** 2024-03-11

**Authors:** Zhao Lin, Megan E. Roche, Víctor Díaz-Barros, Marina Domingo-Vidal, Diana Whitaker-Menezes, Madalina Tuluc, Guldeep Uppal, Jaime Caro, Joseph M. Curry, Ubaldo Martinez-Outschoorn

**Affiliations:** 1 Department of Medical Oncology, Sidney Kimmel Cancer Center, Thomas Jefferson University, Philadelphia, PA 19107, USA.; 2 Immunology, Microenvironment & Metastasis Program, Wistar Institute, Philadelphia, Pennsylvania, USA.; 3 Department of Pathology, Anatomy and Cell Biology, Sidney Kimmel Cancer Center, Thomas Jefferson University, Philadelphia, PA 19107, USA.; 4 Cardeza Foundation for Hematologic Research, Department of Medicine, Sidney Kimmel Cancer Center, Thomas Jefferson University, Philadelphia, PA 19107, USA.; 5 Department of Otolaryngology-Head and Neck Surgery, Sidney Kimmel Cancer Center, Thomas Jefferson University, Philadelphia, PA, USA.

**Keywords:** oxidative stress, miR-200c, chromatin modification, senescence, NFκB-HIF signaling, cancer immunology and immunotherapy

## Abstract

Mesenchymal-epithelial plasticity driving cancer progression in cancer-associated fibroblasts (CAFs) is undetermined. This work identifies a subgroup of CAFs in human breast cancer exhibiting mesenchymal-to-epithelial transition (MET) or epithelial-like profile with high miR-200c expression. MiR-200c overexpression in fibroblasts is sufficient to drive breast cancer aggressiveness. Oxidative stress in the tumor microenvironment induces miR-200c by DNA demethylation. Proteomics, RNA-seq and functional analyses reveal that miR-200c is a novel positive regulator of NFκB-HIF signaling via COMMD1 downregulation and stimulates pro-tumorigenic inflammation and glycolysis. Reprogramming fibroblasts toward MET via miR-200c reduces stemness and induces a senescent phenotype. This pro-tumorigenic profile in CAFs fosters carcinoma cell resistance to apoptosis, proliferation and immunosuppression, leading to primary tumor growth, metastases, and resistance to immuno-chemotherapy. Conversely, miR-200c inhibition in fibroblasts restrains tumor growth with abated oxidative stress and an anti-tumorigenic immune environment. This work determines the mechanisms by which MET in CAFs via miR-200c transcriptional enrichment with DNA demethylation triggered by oxidative stress promotes cancer progression. CAFs undergoing MET trans-differentiation and senescence coordinate heterotypic signaling that may be targeted as an anti-cancer strategy.

## INTRODUCTION

Metastasis and treatment refractoriness are determinants of cancer-related mortality [1]. Metastasis is a multistage scenario that requires carcinoma cells to escape from the primary tumor, travel in the circulation, seed distant organs and grow [1]. The tumor microenvironment (TME) influences each of the steps in carcinoma cell dissemination [2]. In fact, a variety of stromal cells recruited to tumors promote primary tumor growth and metastatic dissemination [3–5]. Cancer-associated fibroblasts (CAFs) reprogram the extracellular matrix (ECM), metabolism and immune function to promote cancer progression and treatment resistance [6, 7].

A canonical myofibroblast state, termed pro-tumorigenic CAFs manifest with hierarchical characteristic features, including inflammatory signaling, metabolic derangements and immunosuppression [6, 7]. Oxidative stress and glycolytic metabolism in fibroblasts drive this CAF phenotype [8]. HIF and NFκB signaling frequently activated in CAFs lead to inflammatory-metabolic reprogramming with upregulation of the monocarboxylate transporter 4 (MCT4), lactate release, chemokine and cytokine recruitment, and loss of the lipid raft signal transduction protein caveolin-1 (CAV1) [9, 10]. However, the fundamental mechanisms regarding heterogeneity of CAFs such as mesenchymal-to-epithelial transition (MET)/epithelial–like phenotype and whether they are sufficient for cancer progression have not been explored.

Senescent fibroblasts promote tumor growth, invasion, and metastasis through senescence-associated secretory phenotype (SASP), which involves the secretion of pro-inflammatory cytokines, chemokines, growth factors, and extracellular matrix remodeling enzymes [11–13]. Additionally, senescent fibroblasts are involved in the recruitment and activation of immune cells, leading to a shift towards an immunosuppressive tumor microenvironment [12]. Although the secretory phenotype of senescent fibroblasts contributing to a pro-tumorigenic TME has been studied, the mechanisms of senescence initiation in a paracrine fashion by adjacent carcinoma cells and its relationship with MET are poorly understood.

Trans-differentiation driving cancer aggressiveness is a common phenomenon [5]. In carcinoma cells, epithelial-mesenchymal plasticity (EMP) encompassing the epithelial-to-mesenchymal transition (EMT) and its reverse MET, play key roles in metastatic dissemination of cancer [14–16]. Also, EMP is linked to stemness [17] and epigenetic reprogramming [18]. Increasing evidence supports the notion that multiple types of cells foster cancer initiation and progression. Not only stem cell-like cancer cells with EMT features, but also carcinoma cells undergoing MET or even intermediate states can promote aggressiveness [19–21]. However, the contribution of fibroblast stemness and MET or trans-differentiation to cancer aggressiveness is unknown.

MicroRNAs (miRs) are 20-22 nucleotide non-coding RNAs that mainly silence genes via targeting their 3'UTRs, and are involved in multiple cellular processes, including development, proliferation, and apoptosis [22]. Specifically, studies have shown that miR-200c and miR-205 induce MET by downregulating ZEB1/2 and upregulating E-cadherin [23–26]. Research to date has not addressed the role of miR-200c in CAFs as a driver of cancer aggressiveness. Here, we determine that reprogramming fibroblasts via miR-200c undergoing MET with a senescent phenotype triggered by oxidative stress is sufficient to drive breast cancer (BRCA) progression with a pro-tumorigenic CAF profile.

## RESULTS

### MET/epithelial-like profile in CAFs concatenates with high miR-200c and its promoter hypomethylation in human BRCA

**Figure 1:**
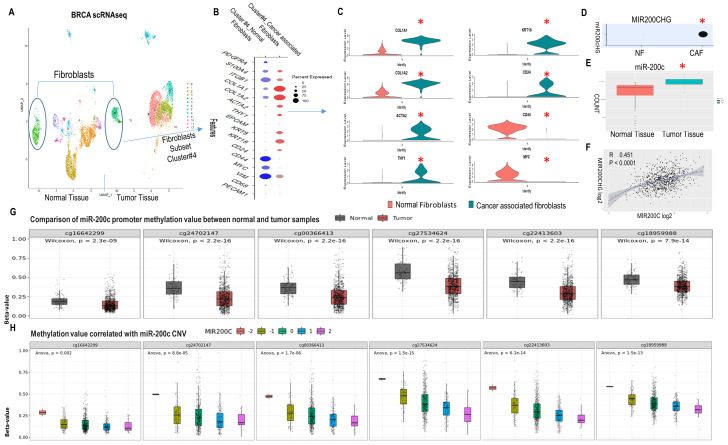
FIGURE 1: MET profile of CAFs in human breast cancer linked high miR200c expression to hypomethylation of the miR-200c promoter. **(A)** UMAP visualization of assigning normal fibroblast and CAF identity to cluster 4 and 5 in normal breast tissue (NT) and breast cancer tumor tissue (TT) by scRNAseq. Circled clusters are fibroblasts. All cell type profiles are listed in SFig. 1A. **(B)** Expression of markers in fibroblast cluster 4 from NT and TT by DotPlot. **(C)** VinPlot of expression of collagen, activated fibroblast, EMP and stemness markers in cluster 4 from NT and TT. *Seurat adjusted p < 0.05. **(D)** MIR200CHG level in cluster 4 from NT and TT. **(E)**Level of miR-200c in NT and TT from the TCGA breast cancer miRNAseq dataset. **(F)** Correlation between MIR200CHG and miR-200c from the TCGA breast cancer dataset. **(G)** Methylation status of miR-200c promoter region. Six sites are hypo-methylated in the tumor versus normal group. **(H)**Plot of the correlation between miR200c CNV and promoter methylation revealing a negative correlation. (A-D) are based on publicly available scRNA-seq data sets (GEO accession code GSE161529: GSM4909254 and GSM4909296). Data analysis was done in R using the Seurat package v4.3 with default parameters, padj<0.05.

Single cell RNA sequencing (scRNASeq) allows assessment of intratumoral heterogeneity. Using Seurat, we analyzed relevant markers of fibroblast clusters in publicly available scRNASeq data from human BRCA and normal breast tissue samples (GEO accession code GSE161529: GSM4909254 and GSM4909296) [27]. The fibroblast subclass was validated based on high expression of the fibroblast markers PDGFRA, S100A4, ITGB1 and COL1A1. The CAF subgroup was classified based on ACTA2, THY1 and collagen related genes COL1A1, COL1A2 (**Fig. 1A-C** and SFig. 1A). Human cluster gene signatures revealed that in the BRCA CAF cluster 4, COL1A1, COL1A2, ACTA2, THY1 and epithelial markers (CD24, KRT8, KRT18) increased, while mesenchymal markers (VIM, CD44) and stemness markers (MYC, CD44) decreased compared to the fibroblast cluster 4 of normal breast tissue (**Fig. 1B, C**). These findings were validated in another publicly available scRNASeq data of BRCA (E-GEOD-75688) [28] (SFig. 1B-F). These observations support the existence of MET/reduced stemness in CAFs of human BRCA.

The existence of MET in CAFs raised the question of whether miRs and epigenetic modifications were the drivers. First, MIR200CHG was highly expressed in BRCA CAFs and absent in normal breast fibroblasts (**Fig. 1D**). MIR200CHG is a long non-coding RNA, and miR-200c is located within its intron. Further studies of the TCGA BRCA dataset revealed that miR-200c was highly expressed in BRCA in contrast to normal breast tissue (**Fig. 1E**), and there was a positive correlation between MIR200CHG and miR-200c (**Fig. 1F**). Second, the promoter of miR-200c has six CpG enriched regions that are potential sites for cytosine methylation (SFig. 2A). All six CpG sites were significantly hypo-methylated in the tumor versus the normal breast group of the TCGA BRCA dataset (**Fig. 1G**, SFig. 2B). Moreover, miR-200c copy number variation (CNV) was negatively correlated to promoter CpG methylation (**Fig. 1H**). Hence, these data from human BRCA revealed that CAFs with a MET phenotype exist and high miR-200c levels correlates with DNA de-methylation of its promoter.

**Figure 2:**
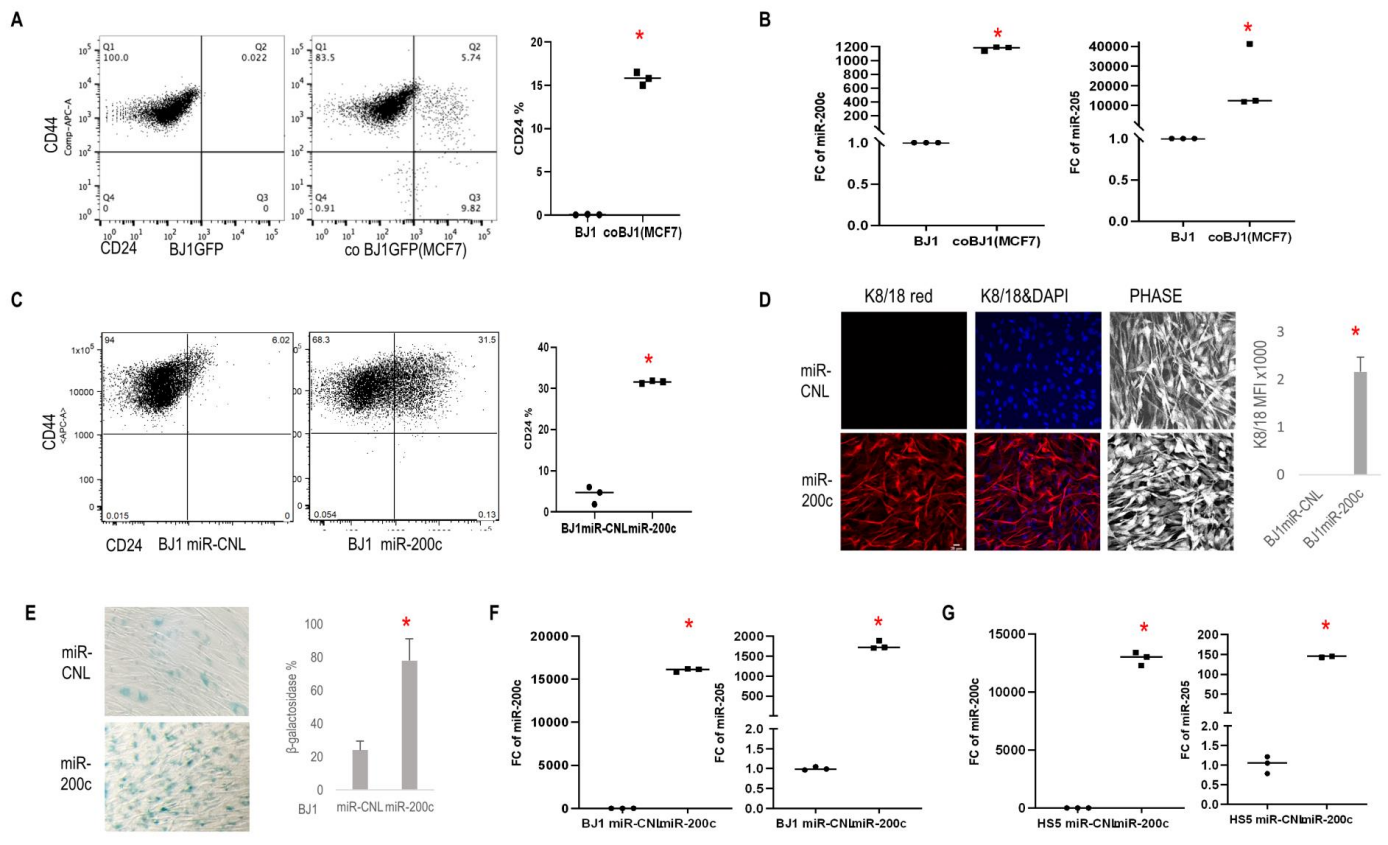
FIGURE 2: Effects of carcinoma cells and miR-200c on fibroblasts. **(A)** CD24 and CD44 expression patterns by flow cytometry in BJ1 fibroblasts under homotypic and co-culture conditions with MCF7 carcinoma cells. **(B)** Fold change of miR-200c and miR-205 expression in BJ1 fibroblast cells co-cultured with carcinoma cells compared to homotypic culture. **(C)** Flow plots showed CD24 and CD44 level change in BJ1 cells overexpressing miR-200c and scramble control. **(D)** Images of cell morphology and K8/18 (Red) expression of BJ1 with precursor miR-200c expression and scramble control (scale bar: 20 μm). **(E)** Senescence β-galactosidase staining in BJ1 cells overexpressing miR-200c and scramble control. **(F, G)** Fold change in miR-200c and miR-205 expression in BJ1 and HS5 fibroblast cells with overexpression of miR-200c or control. *p<0.05.

To assess if crosstalk with carcinoma cells could induce reprogramming of fibroblasts, a co-culture system with carcinoma cells and fibroblasts was employed [29]. First, a subclass of cocultured fibroblasts increased CD24 (epithelial reprogramming marker) and reduced CD44 (marker of cellular differentiation) expression (**Fig. 2A**). Next, fibroblasts under coculture conditions displayed high miR-200c and miR-205 levels (**Fig. 2B**). Using BJ1 fibroblasts stably overexpressing miR-200c (BJ1miR-200c), we observed similar effects with upregulation of CD24 and downregulation of CD44 compared to control (miR-CNL) (**Fig. 2C**) and a subset of fibroblasts displayed an epithelial-like phenotype with keratin 8/18 (K8/18) expression (**Fig. 2D**). Meanwhile miR-200c significantly induced senescence β-galactosidase (**Fig. 2E**). It is well-known that miR-200c and miR-205 trigger MET, hence we assessed the effects of overexpressing miR-200c on miR205 expression in BJ1 and HS5 fibroblasts and found that miR-200c drove miR-205 expression (**Fig. 2F, G**).

### Reprogramming fibroblasts via miR-200c induces senescence, histone deacetylation and demethylation, a reduced stemness signature, and activation of NFκB and HIF signaling with inflammatory-glycolytic features

**Figure 3:**
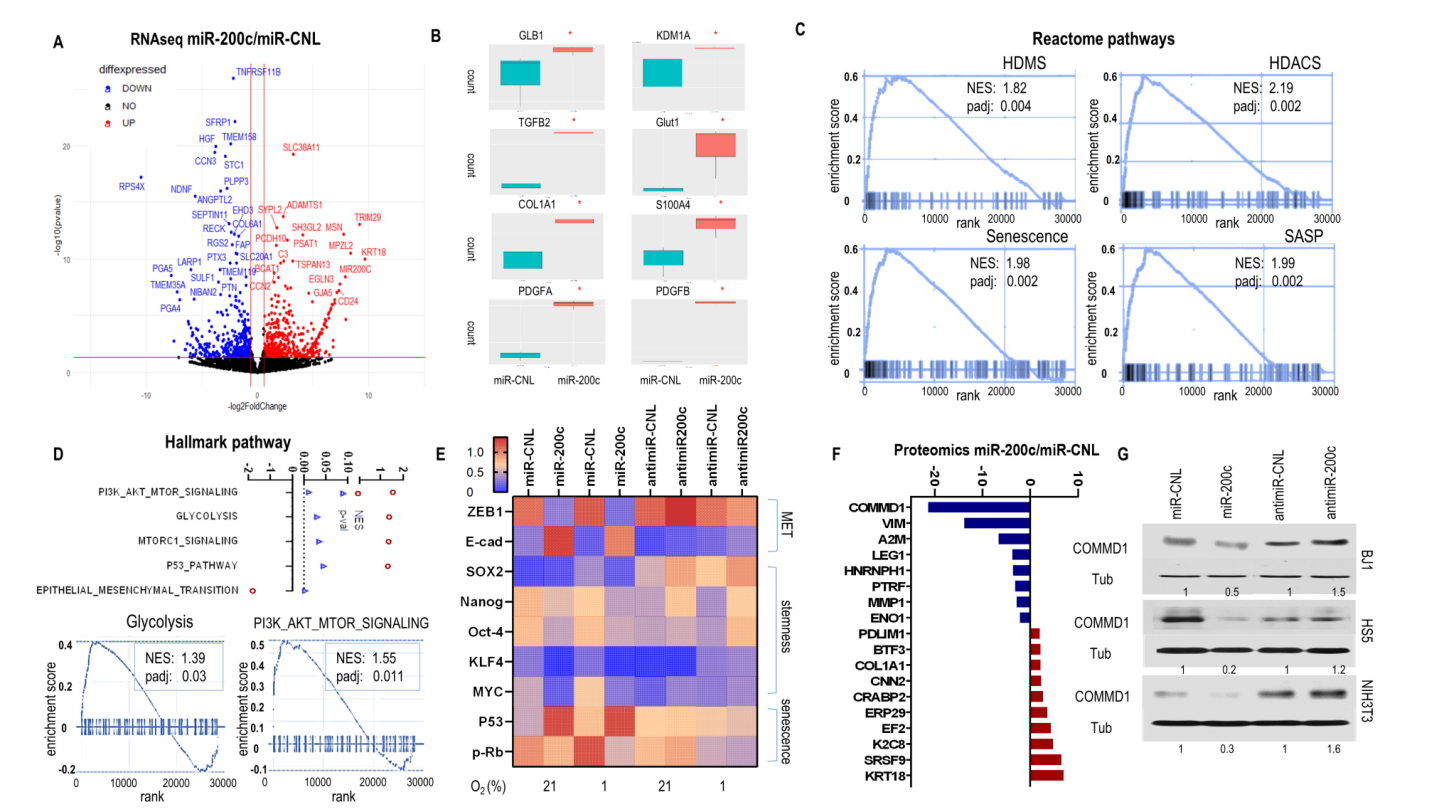
FIGURE 3: Effects of fibroblast miR-200c on MET, senescence and CAF phenotype. (A-D) RNAseq data for BJ1miR-200c versus control. **(A)** Volcano plot showed the most differentially expressed genes. **(B)** Boxplots for the relevant markers expression of senescence, HDMs, cytokine, glycolysis and growth factors. **(C)** GSEA plot enrichment of Reactome pathways driven by miR200c **(D)** GSEA of Hallmark pathways driven by miR200c. **(E)** Protein levels of BJ1 cells overexpressing or inhibiting miR-200c and control under normoxic and hypoxic conditions. **(F)** Barplot from proteomic assay showing genes with fold change >2 between BJ1miR-200c and control. **(G)** COMMD1 level in fibroblasts with miR-200c overexpression or inhibition.

To explore molecular biological pathways due to miR-200c enrichment, we performed whole transcriptome RNAseq with BJ1miR-200c and miR-CNL. DEseq2 analysis identified 3355 genes including 1848 upregulated and 1507 downregulated in cells overexpressing miR-200c in contrast to control. A volcano plot displayed the most differentially expressed genes (**Fig. 3A**). Epithelial markers (KRT18, CD24), senescence marker GLB1, histone demethylase KDM1A, an inflammatory cytokine TGFB2, a glycolysis marker GLUT1, fibroblast markers (S100A4, COL1A1) and growth factors (PDGFA, PDGFB) significantly increased with high miR-200c (**Fig. 3A, B**, SFig. 3A). Gene Set Enrichment Analysis (GSEA) revealed that miR-200c upregulated senescence signatures with histone deacetylation (HDACS) and demethylation (HDMS) in Reactome pathways (Fig.3C, SFig.3B). HDAC-containing complexes cooperate with histone demethylases (HDMs) to induce transcriptional repression. MiR-200c drove MET (reduced EMT), glycolysis, P53 and PI3K-AKT-MTOR pathways in Hallmark Pathway assessment (**Fig. 3D**). Glycolysis, P53 and MTOR upregulation are associated with a senescent phenotype. PI3K-AKT-MTOR pathway is critical to stem cell proliferation, metabolism and differentiation. Concurrently, protein level analysis showed that miR-200c induced an MET profile with ZEB1 loss and E-cadherin gain, abrogated a reduced iPSC/stemness phenotype with MYC, OCT4, SOX2, KLF4 and NANOG suppression, and induced a senescent phenotype with P53 upregulation and p-Rb downregulation (**Fig. 3E**). Conversely, anti-miR-200c induced an iPSC/stemness profile with MYC, OCT4, SOX2 and NANOG upregulation (**Fig. 3E**, SFig. 3C). These data demonstrated that reprogramming fibroblasts via miR-200c undergoing MET/senescence with abnormal chromatin change and reduced stemness generated an inflammatory-glycolytic signature.

To further investigate the potential regulatory mechanisms of miR-200c, a proteomic assay was performed with BJ1miR-200c versus control. The most differentially expressed (FC >2) genes are shown in **Fig. 3F**. Classical MET comprising high keratin 8/18 (KRT8/18) and low vimentin (VIM) was observed in BJ1miR-200c. Also, lower caveolar protein PTRF was observed in BJ1miR-200c, which is a feature of CAFs. Particularly, COMMD1 was dramatically diminished (**Fig. 3F**). We confirmed the regulation of COMMD1 by miR-200c, since overexpression of miR-200c in various fibroblasts repressed COMMD1 and anti-miR-200c induced it (**Fig. 3G**). Intriguingly, COMMD1 and H3K4me3 levels are decreased in both fibroblast and cancer cells under co-culture conditions relative to homotypic culture (SFig. 4A).

**Figure 4:**
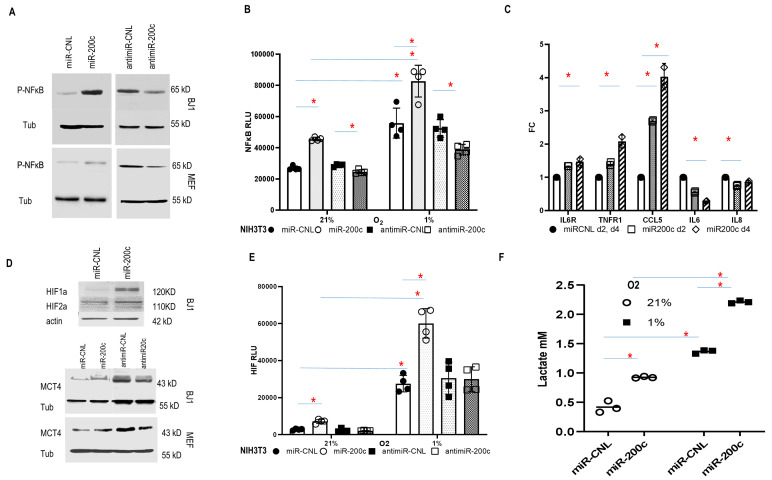
FIGURE 4: Effect of miR-200c overexpression in fibroblasts on NFκB and HIF pathways. **(A)** Phospho-NFκB p65 expression of fibroblasts with miR-200c downregulation or overexpression. **(B)** NFκB activity in NIH3T3 NFκB luciferase reporter cells with miR-200c overexpression or downregulation under normoxic (21% O2) and hypoxic (1% O2) conditions (Random Luciferase Units-RLU). *p<0.05. **(C)** Cytokine, chemokine and receptor expression profile in fibroblast conditioned media. BJ1 F200c or control were cultured in serum-free DMEM media. The levels of various factors in the cell-free culture media were measured by antibody arrays at day 2 and 4. Data was showed as fold change ± SD. *p<0.05. **(D)** HIF and MCT4 expression in fibroblasts with miR-200c modulation. **(E)** HIF activity in NIH3T3 HIF luciferase reporter cells with miR-200c overexpression or downregulation under normoxic and hypoxic conditions. *p<0.05. **(F)** Lactate level in BJ1miR-200c and control under normoxic and hypoxic conditions. *p<0.05.

The COMMD protein family has been identified as a negative regulator of NFκB and HIF signaling, which are two key inflammatory pathways [30–33]. NFκB activity was induced by miR-200c and inhibited by anti-miR-200c (**Fig. 4A**). Accordingly, in established NIH3T3 NFκB luciferase reporter fibroblasts, miR-200c increased NFκB activity in normoxia and to a greater degree under hypoxia, while anti-miR-200c reversed it (**Fig. 4B**). NFκB can mediate inflammatory signaling and facilitate an immune-inflammatory milieu [10]. Stromal cells can be reprogrammed by carcinoma cells to secrete pro-tumorigenic growth factors, and cytokines [34]. To better understand whether miR-200c could reprogram the cytokine profile, serum-free cell culture media from BJ1 miR-200c and control cells were screened for the levels of 40 inflammatory mediators after two and four days. The factors IL6R, TNFR1, CCL5, IL6 and IL8 were expressed differentially (**Fig. 4C**). Notably, the level of the chemokine CCL5 increased considerably over time. CCL5 can be induced by NFκB and senescence related to SASP, which activates immune-regulatory and inflammatory processes, and contributes to metastasis and chemo-resistance in BRCA [35]. Also, CCL5 inhibited CAV1 expression in BJ1 cells (SFig. 4B). Predictably, miR200c overexpression in fibroblasts repressed CAV1, whereas anti-miR200c induced CAV1 (SFig. 4C, D). Moreover, the HIF antagonist PT2399 upregulated CAV1 in control BJ1 cells (SFig. 4D). Loss of CAV1 is a critical feature of CAFs [10].

HIF signaling is a major driver of tumor progression [36]. To determine the contribution of miR-200c to HIF activity in fibroblasts, HIF and its downstream target MCT4 were assessed. They were upregulated by miR-200c, while anti-miR-200c suppressed MCT4 expression (**Fig. 4D**). Accordingly, in HIF reporter cells, miR-200c triggered HIF activity in normoxia and to a greater level under hypoxia, while anti-miR-200c reduced HIF activity (**Fig. 4E**). Moreover, hypoxia induced miR-200c expression in fibroblasts (SFig. 4E). Finally, miR-200c induced glycolysis with lactate accumulation (**Fig. 4F**). In sum, miR-200c drove inflammatory-glycolytic reprogramming with HIF and NFκB activation in fibroblasts.

### MiR-200c in fibroblasts promoted cancer cells aggressiveness

**Figure 5:**
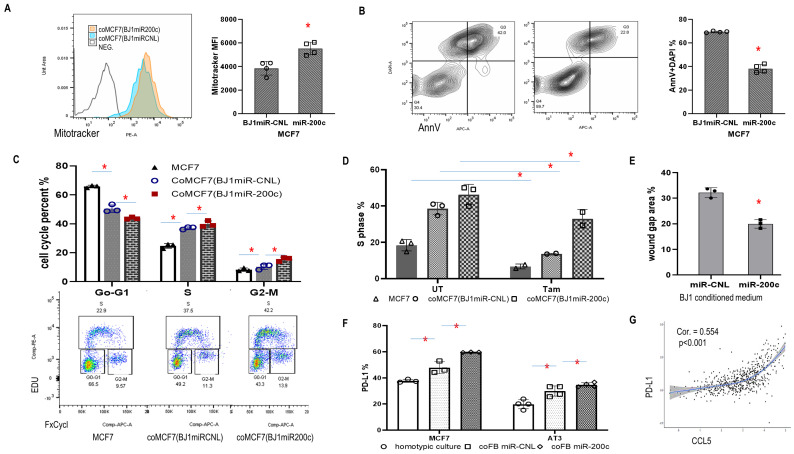
FIGURE 5: Effects of fibroblast miR-200c *in vitro* on features of cancer aggressiveness. **(A-C)** Flow data showed functional mitochondrial mass, apoptosis and proliferation in cancer cells co-cultured with F200c. **(D)** S phase of MCF7 cells in homotypic culture or co-culture with miR-200c BJ1 fibroblasts and controls, with and without Tamoxifen treatment for 24 hours. **(E)** Wound healing. Wound gap area of MCF7 cells exposed to conditioned media from BJ1 miR-200c or control at time points 0 and 16 hours post-exposure. **(F)** PD-L1 expression in MCF7 and AT3 cancer cells under homotypic and co-culture with miR200c fibroblasts or controls. *p<0.05. **(G)** Correlation between CCL5 and PD-L1.

High mitochondrial mass with resistance to apoptosis is a common feature of carcinoma cells. Here, we used a coculture system to investigate the effects of miR-200c in fibroblasts on the mitochondrial activity of carcinoma cells. First, MCF7 carcinoma cells co-cultured with BJ1miR-200c displayed higher mitotracker orange signal (**Fig. 5A**). Second, high miR-200c in fibroblasts inhibited MCF7 apoptosis and promoted MCF7 proliferation (**Fig. 5B, C**). Third, miR-200c protected MCF7 cells from the antiestrogen tamoxifen (**Fig. 5D**). Fourth, serum-free conditioned media from BJ1miR-200c induced the migration of MCF7 cells to a greater extent than controls with a reduced wound gap area (**Fig. 5E**).

Intriguingly, co-culture with miR-200c overexpressing fibroblasts induced PD-L1 expression in MCF7 and AT3 carcinoma cells (**Fig. 5F**). PD-L1 is a critical component of the immune checkpoint that suppresses anticancer immunity. Since both PD-L1 and CCL5 were upregulated by miR-200c, the TCGA BRCA database was studied and we found a positive correlation between CCL5 and PD-L1 (**Fig. 5G**). These data supported downstream effects of fibroblast miR-200c on cancer progression and immune reprogramming.

### *In vivo* miR-200c in fibroblasts induced tumor growth, metastasis and resistance to chemo-immunotherapy

**Figure 6:**
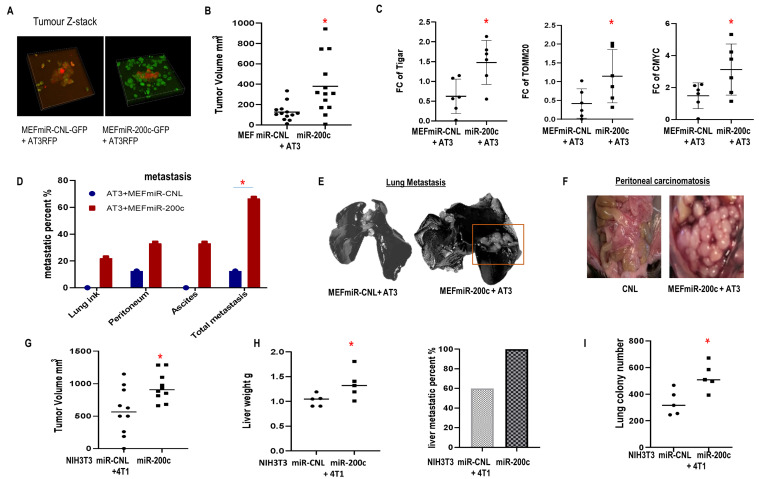
FIGURE 6: Effect of miR-200c overexpression in fibroblasts on primary breast cancer tumor growth and metastasis. **(A-F)** Co-injection of AT3-RFP cells with MEFs-GFP miR-200c or controls. Mice were sacrificed 6 weeks post-injection. **(A)** Tumor Z-stack images. **(B)** Tumor volume. **(C)** Immunoblot to assess the metabolic markers in the generated tumors. **(D)** Prevalence of metastatic disease. **(E, F)** Representative images for lung India ink staining and peritoneal metastasis are shown. **(G-I)** Co-injection of 4T1 cells and miR-200c NIH3T3 fibroblasts or controls. Mice were sacrificed 18 days post-co-injection. **(G)** Tumor volume. **(H)** Liver weight and metastatic percent. **(I)** Lung metastatic colonies. 6-TG resistant lung colonies quantified using image J.

Co-injection of breast carcinoma cells with fibroblasts overexpressing miR-200c or control were performed. The co-injected MEFs were still present in the tumors at the time of sacrifice (**Fig. 6A**), MEFs with miR-200c overexpression co-injected with AT3 triple negative breast carcinoma cells promoted tumor growth versus controls (**Fig. 6B**). Immunoblots showed that miR-200c in MEFs *in vivo* induced expression of proteins related to metabolic reprogramming including TIGAR, MYC and TOMM20 (**Fig. 6C**). 66.7% of mice from miR200c group developed metastases comprising the lungs, peritoneal implants and malignant ascites, whereas only 12.5% of control mice had metastatic disease (p<0.05) (**Fig.6D-F**). Specifically, metastases visualized with India ink staining of lungs and ascites only occurred in the miR-200c group (**Fig. 6E, F**).

Likewise, when BALB/c mice were inoculated with syngeneic triple negative 4T1 breast carcinoma cells and syngeneic NIH3T3 F200c or control, similar results were observed. MiR-200c in NIH3T3 cells triggered tumor growth and metastatic dissemination to multiple distant organs including livers and lungs (**Fig. 6G-I**). Also, MCF7 cells co-injected with BJ1 overexpressing miR-200c generated larger tumors (SFig. 5A).

**Figure 7:**
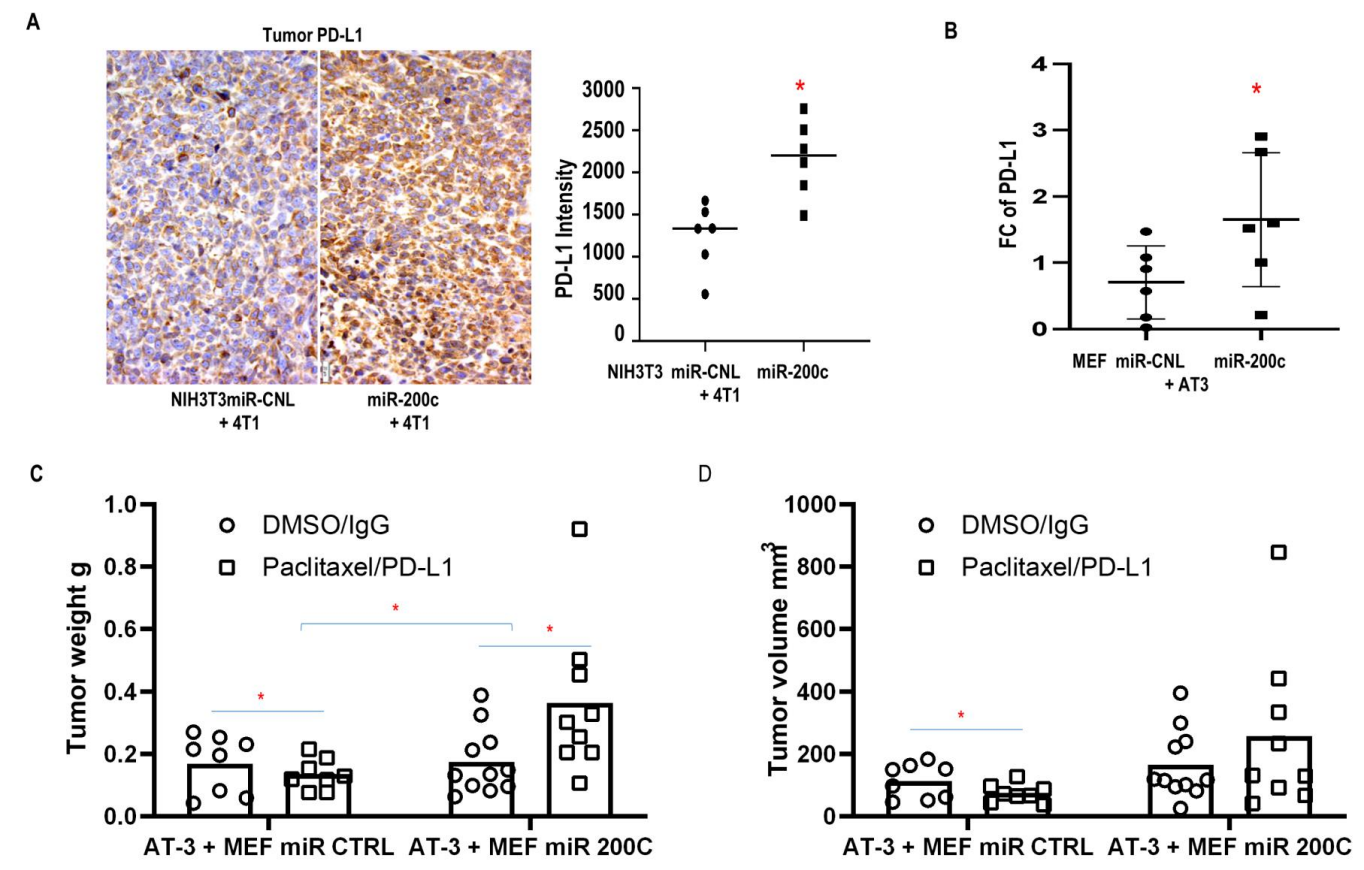
FIGURE 7: Effect of miR-200c in fibroblasts on resistance to chemo-immuno-therapy. **(A)** Representative PD-L1 staining in tumor sections. Co-injection of 4T1 cells and miR-200c NIH3T3 fibroblasts or controls (scale bar: 20μm). Immunohistochemistry quantification for PD-L1 in random units as per ImageJ quantification. **(B-D)** Co-injection with AT3 cells and MEFs miR-200c or control. **(B)** PD-L1 immunoblot. **(C, D)** Tumor growth with chemo-immunotherapy. Mice were treated by IP with Paclitaxel and anti-PD-L1 versus DMSO and control IgG after tumor implantation.

In addition, MiR-200c in fibroblasts promoted resistance to immune checkpoint blockade. Mice carrying tumors composed of syngeneic 4T1 cells and NIH3T3 F200c developed more hepatosplenomegaly with inflammatory features including larger spleens with hyperplasia of the red pulp containing expanded megakaryocytes (SFig. 5B, C). Moreover, admixing carcinoma cells with F200c *in vivo* enhanced PD-L1 expression, which promoted cancer immune evasion (**Fig. 7A, B**).

Importantly, MEFs overexpressing miR-200c co-injected with AT3 cells induced resistance to the combination of paclitaxel and PD-L1 inhibition, while tumors with control fibroblasts were sensitive to the combined immuno-chemotherapy (**Fig. 7C, D**). The combination of Paclitaxel and anti-PD-L1 was approved by the FDA as an immunotherapy regimen for triple negative BRCA.

Taken together, these *in vivo* experiments demonstrated that miR-200c in fibroblasts caused metastatic disease and treatment resistance with an inflammatory-metabolic and immunosuppressive phenotype.

### MiR-200c inhibition in fibroblasts reduces tumor growth

**Figure 8:**
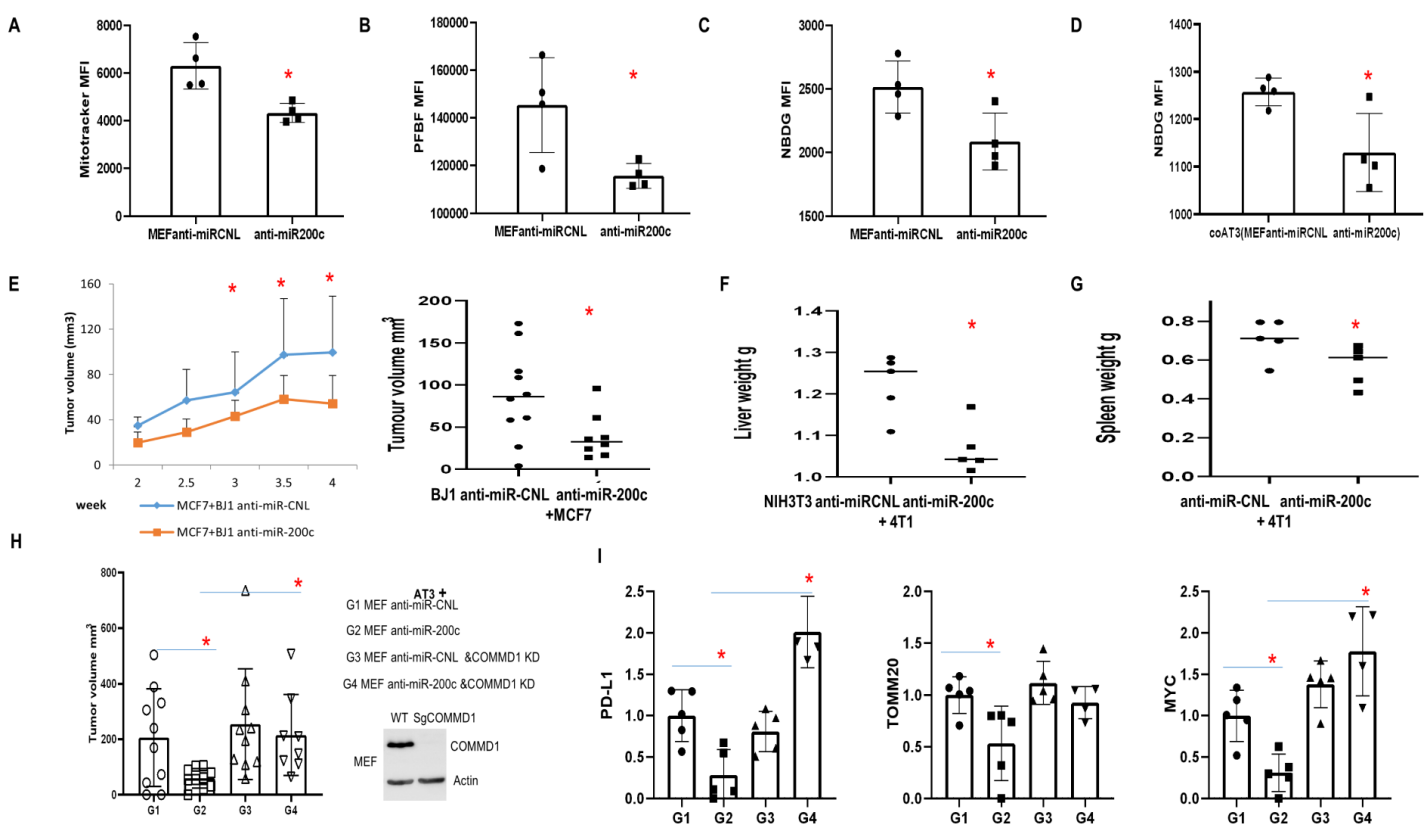
FIGURE 8: Effect of anti-miR-200c and COMMD1 KD in fibroblasts on primary breast cancer tumor growth and metastasis. **(A-C)** Flow data showing functional mitochondrial mass, PFBF and NBDG in MEF overexpressing anti-miR-200c versus control. **(D)** Flow data showing NBDG in AT3 cancer cells co-cultured with fibroblasts overexpressing anti-miR-200c versus control. **(E)** MCF7 cells co-injected with BJ1 anti-miR-200c or control. Tumor volume was measured at over time and at time of sacrifice (n=8-10, *p<0.05). **(F, G)** Liver and Spleen weight from mice co-injected with 4T1 and NIH 3T3 cells anti-miR-200c or control. (n=5-13, *p<0.05). **(H, I)** Co-injection of AT3 cells with MEFs with COMMD1 KD/WT overexpressing anti-miR-200c or controls. Mice were sacrificed 24 days post-injection. (H)Tumor volume. (n=8-10, *p<0.05). (I)Immunoblot to assess PD-L1, TOMM20 and MYC in the generated tumors.

Anti-miR-200c reduced functional mitochondrial mass in fibroblasts, H_2_O_2_ and glucose uptake (**Fig. 8A-C**). Intriguingly, anti-miR-200c in fibroblasts reduced glucose uptake of carcinoma cells under co-culture conditions (**Fig. 8D**). *In vivo* studies revealed that anti-miR-200c in BJ1 fibroblasts suppressed primary tumor growth (**Fig. 8E**). Furthermore, anti-miR-200c in NIH3T3 abrogated hepatosplenomegaly (**Fig. 8F, G**).

Likewise, anti-miR-200c MEFs co-injected with AT3 cells generated smaller tumors in contrast to controls (**Fig. 8H**). Interestingly, COMMD1-KD in the context of anti-miR-200c rescued tumor growth (**Fig. 8H**). Immunoblots revealed that anti-miR-200c in MEFs reduced PD-L1, MYC and TOMM20 expression *in vivo* (**Fig. 8I**), which is the opposite effect to that observed with miR-200c overexpression.

**Figure 9:**
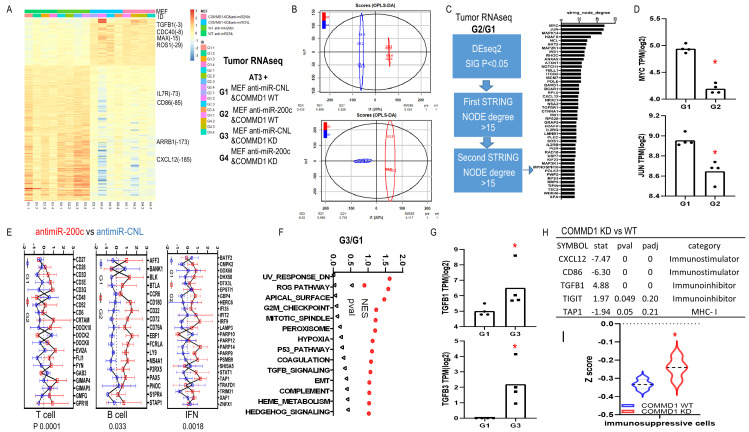
FIGURE 9: Transcriptional effect of anti-miR-200c and COMMD1 KD in fibroblasts on tumor growth *in vivo*. **(A-H)** RNAseq data for mice tumors of co-injection with AT3 and MEF anti-miR-200c/COMMD1 KD versus control. **(A)** Heatmap showed differentially expressed genes in four groups. **(B)** OPLS-DA of G2 versus G1 and G3 versus G1. **(C)** G2 versus G1. Schematic and barplot showed the differentially expressed genes regarding highly connected networks. **(D)** Fold change of MYC and JUN in G2 versus G1. **(E)** Plot for immune assay of antimiR-200c vs control group. **(F)** GSEA hallmark pathways in G3 versus G1. **(G)** Fold change of TGFB1, TGFB3 expression in G3 versus G1. (H)Gene alteration related to check point effect in COMMD1 KD vs WT group. **(I)** Immunophenoscore analysis comparing immunosuppressive cells in COMMD1 KD vs WT group.

To explore molecular biological pathways for downregulation of miR-200c and COMMD1, we performed RNAseq with these four groups of tumor tissues. Analyses revealed strong clustering based on COMMD1 expression (**Fig. 9A, B**). Based on DEseq2 and two rounds of string analyses, 47 genes were identified in the anti-miR-200c group versus control (**Fig. 9C**). All the altered genes were assessed to define the central node of such potential protein–protein interactions (PPI) as well as the associated cellular functions. The interaction network revealed that MYC had the largest node degree, which suggested it is central to the PPI network associated with miR-200 inhibition in fibroblasts. The zooming analysis revealed transcriptional repression as the major function involved in the MYC-interacting proteins. In addition, JUN had the second largest node degree and was assigned to cell differentiation and cell cycle inhibition (**Fig. 9D**). The immune signature of anti-miR-200c groups was consistent with enhanced anti-cancer immune response with enriched T cell, B cell and IFN signatures. In COMMD1-KD versus wild type group (**Fig. 9E**), GSEA Hallmark pathway analyses revealed higher ROS (reactive oxygen species) and TGFB signatures with TGFB1 and TGFB2 upregulation (**Fig. 9F, G**). Furthermore, an immune checkpoint screen and immunophenoscore analysis displayed immunosuppressive traits in the COMMD1-KD group (**Fig. 9H, I**). Together, these data reflect that antimiR200c reduced ROS signaling and activated anti-cancer immune responses while COMMD1-KD upregulated heterotypic pathways including ROS and TGFB with immunosuppression.

### Oxidative stress, DNA demethylation and histone acetylation regulated miR-200c expression

**Figure 10:**
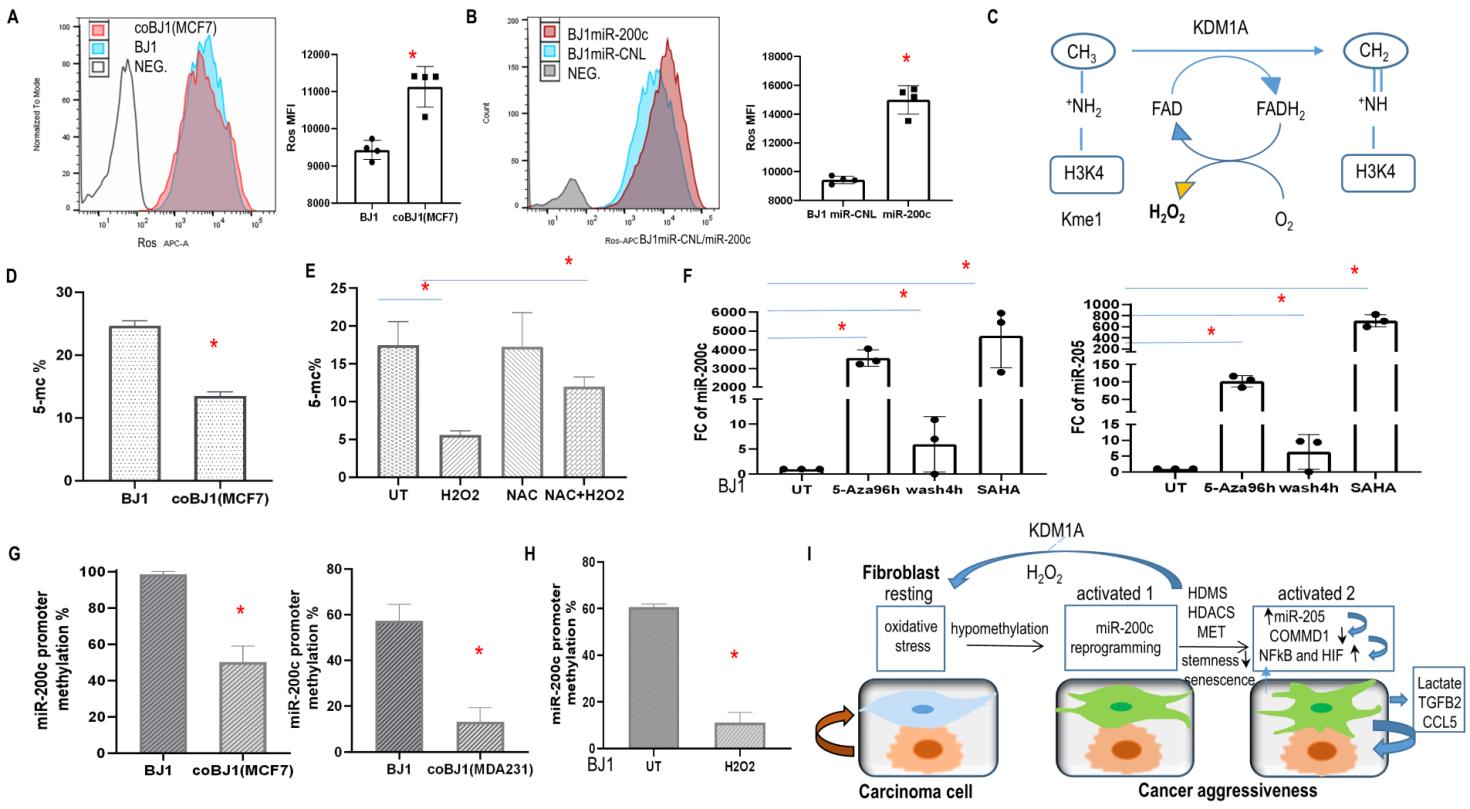
FIGURE 10: Oxidative stress, epigenetic modification regulated miR-200c expression and model of fibroblasts and carcinoma cells cross talk modulated by miR-200c via ROS. **(A)** ROS level in BJ1 cells under homotypic and coculture condition. *p<0.05. **(B)** ROS level in BJ1miR-200c or control. **(C)** Histone demethylation mediated by KDM1A demethylase through an FAD-dependent amine oxidase reaction, releasing one molecule of H_2_0_2_. **(D, E)** Global DNA methylation was measured by 5-mC percentage in sorted co-culture and homotypic BJ1 cells or with redox modulators. **(F)** Fold change of miR-200c and miR-205 in BJ1 fibroblasts exposed to drugs regulating the epigenetic repressive state. **(G, H)** Promoter DNA methylation of miR-200c in fibroblasts. BJ1 cells under homotypic or co-culture condition or exposure to H_2_O_2_ for 4 days_._ *p<0.05. **(I)** Model of fibroblasts and carcinoma cells cross talk regulated by miR-200c. In the TME, ROS causes DNA hypomethylation of fibroblasts, which promotes miR-200c transcription. Reprogramming fibroblasts via miR-200c towards MET/senescence reduces stemness, induces aberrant chromatin changes and histone demethylation mediated by KDM1A demethylase through an FAD-dependent amine oxidase reaction with H_2_0_2_ release. Meanwhile it induces miR-205 and reduces COMMD1 expression along with NFκB and HIF activation. Furthermore, these reprogrammed fibroblasts promote cancer aggressiveness.

NFκB and HIF transcription factors are regulated by redox signaling and ROS [37]. Flow cytometry revealed that carcinoma cells produced more ROS than fibroblasts (SFig. 6A, B) and co-culture induced ROS in fibroblasts (**Fig. 10A**). Interestingly, ROS levels significantly increased in BJ1 cells overexpressing miR-200c (**Fig. 10B**). This is consistent with upregulated KDM1A/LSD1 in fibroblasts with miR-200c and generation of H_2_0_2_ (**Fig. 3B**, **Fig. 10C**).

Next, we studied the effects on miR-200c levels by the redox modulator H_2_O_2_ and the ROS scavenger N-acetyl cysteine (NAC). MiR-200c upregulation was observed with the pro-oxidant H_2_O_2_ and reversed with NAC (SFig. 6C). Moreover, miR-200c expression was elevated in a dose-dependent fashion when BJ1 and HS5 fibroblasts were exposed to H_2_O_2_ (SFig. 6D). Also, COMMD1 was reduced with H_2_O_2_ exposure and rescued by NAC in BJ1 fibroblasts (SFig. 6E). Altogether, these findings indicate that ROS and miR-200c form a positive feedback loop and oxidative stress suppressed COMMD1.

The current study demonstrated that the miR-200c expression of fibroblasts was regulated by DNA methylation as an epigenetic silencing mechanism. DNA methylation maintains EMT programs in carcinoma cells [38]. Reduced genome-wide 5 methyl-cytosines (5-mC) were observed in fibroblasts under co-culture conditions and exposure to H_2_0_2_, whereas NAC could rescue it (**Fig. 10D, E**). To verify whether epigenetic modification modulated the expression of miR-200c and miR-205, HS5 and BJ1 fibroblasts were treated with the DNA cytosine methylation inhibitor 5-Aza-2′-deoxycytidine (5-Aza) and the histone deacetylase inhibitor SAHA. As expected, miR-200c and miR-205 levels increased dramatically with four days of treatment (**Fig. 10F**, SFig. 6F). However, when 5-Aza was washed out for 4 hours, both microRNAs decreased markedly (Fig.10F, SFig.6F). Moreover, gain of CD24 and loss of CD44 ratio were observed with Aza treatment for 4days in hTERT-BJ1 cells (SFig.6G). These observations revealed a direct role of DNA methylation and histone deacetylation on the transcriptional modulation of miR-200c and miR-205 in fibroblasts.

To further investigate the promoter methylation status of miR-200c, we performed comparative bioinformatics coupled with DNA methylation analysis and qPCR. CpG islands, which are CpG dinucleotide-rich regions, often co-localize with promoters, and methylation of the cytosines of the CpG dinucleotides leads to transcriptional silencing^40^. We therefore focused on the promoter region of miR-200c. Sequence analysis revealed two well-defined CpG islands upstream of the miR-200c/141 cluster. Two pairs of primers were generated to detect methylation. As anticipated, methylation repression occurred in fibroblasts under co-culture condition and exposure to H_2_O_2_ (Fig. 10G, H). Hence, miR-200c transcription is regulated by DNA methylation.

## DISCUSSION

**Figure 11:**
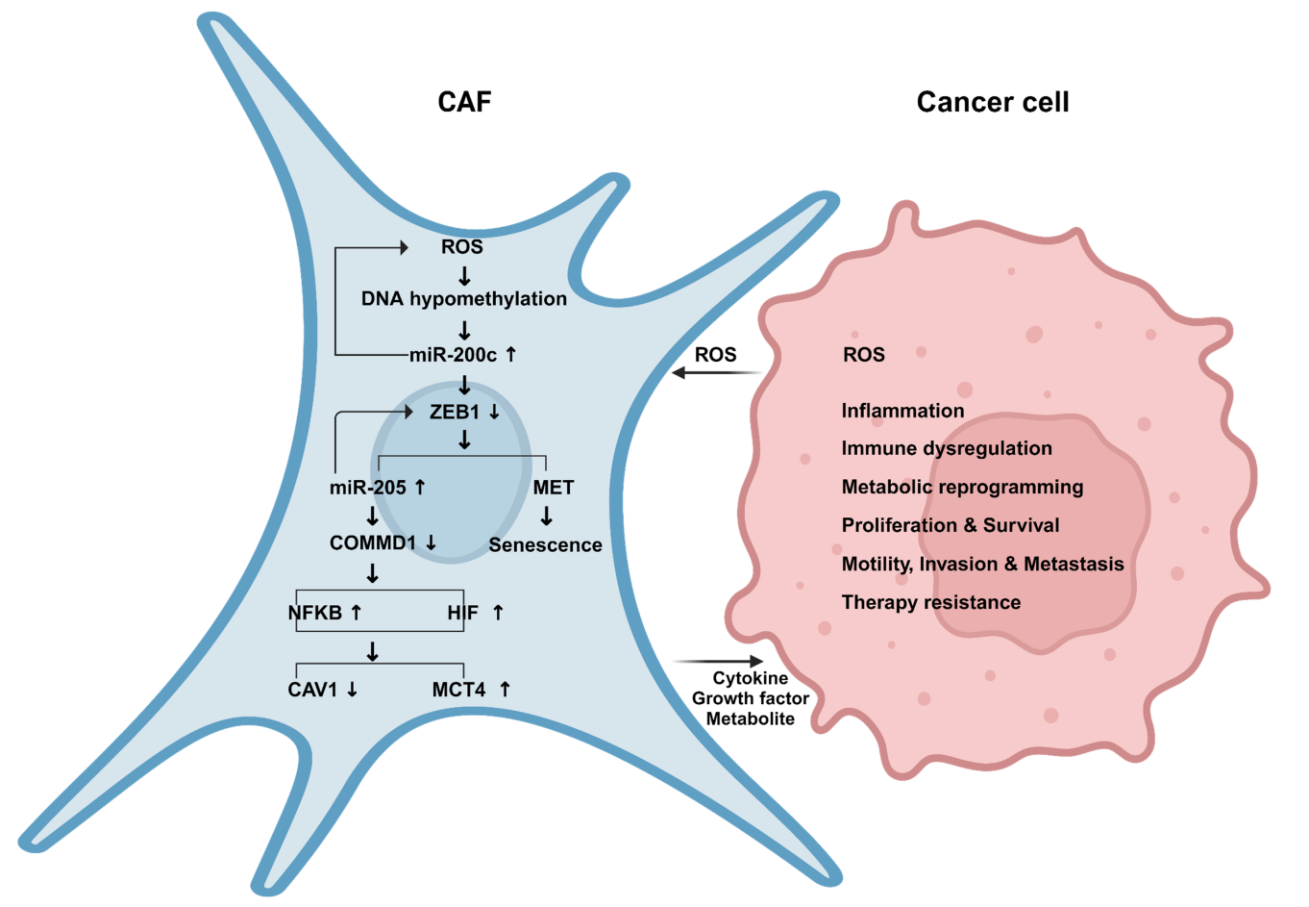
FIGURE 11: Graphical Summary. In the TME, the interaction between fibroblasts and carcinoma cells stimulates oxidative stress with reactive oxygen species (ROS) release. ROS in fibroblasts triggers DNA hypomethylation leading to miR-200c transcriptional enrichment. MET in fibroblasts via miR-200c induces senescence, miR-205 and suppresses COMMD1 that activates NFκB and HIF signaling and recapitulates the phenotype of CAFs with downregulation of CAV1 and upregulation of MCT4 as well as cytokines, growth factors and lactate release. Paramountly, these reprogrammed fibroblasts promote cancer aggressiveness.

This study identifies a novel mechanism of pro-tumorigenic heterogeneity in CAFs (**Fig. 11**). We have deciphered that a subgroup of CAFs in human BRCA undergo a reprogramming process that resembles MET/epithelial-like profile with high miR-200c expression. Reprogramming fibroblasts via miR-200c towards MET/senescence reduces stemness and induces aberrant chromatin changes, especially histone demethylation by KDM1A demethylase with hydrogen peroxide release. We report here that miR-200c in fibroblasts as a novel positive regulator of NFκB-HIF signaling via COMMD1 depletion, recapitulates the cardinal inflammatory-glycolytic features of CAFs and promotes tumor growth, metastasis and resistance to immuno-chemotherapy. Whereas miR-200c inhibition in fibroblasts not only reduces ROS and NFkB-HIF activity but also induces an anti-cancer immune profile and restrains tumor growth. Conversely, COMMD1 suppression abrogates the effects of antimiR-200c.

Mechanistically, we determine that miR-200c in fibroblasts is regulated by epigenetic reprogramming. The crosstalk between cancer cells and fibroblasts induces oxidative stress, which drives DNA demethylation, and in particular, leads to promoter hypo-methylation of miR-200c in fibroblasts with enriched miR-200c transcription. Moreover, we have validated that there is a positive feed loop mechanism whereby miR-200c is sufficient to stimulate oxidative stress via histone demethylation (HDMS) upregulation.

We have discovered that miR-200c promotes trans-differentiation of fibroblasts toward a MET/senescent phenotype with reduced ZEB1, increased E-cadherin and miR-205, and suppressed COMMD1, which is a target gene of miR-205 and inhibitor of NFκB and HIF activity. COMMD1 plays pleiotropic roles in cancer progression modulating oxidative stress, DNA damage response, as well as NFκB and hypoxia mediated transcription [39]. Reprogramming fibroblasts via miR-200c is sufficient to recapitulate the canonical features of CAFs with NFκB and HIF activation comprising loss of CAV1 and gain of GLUT1, MCT4. The current study expands the understanding of cross-activation mechanisms between NFκB and HIF [40, 41].

Metabolic and inflammatory reprogramming are hallmarks of cancer [42]. Here we determine that the senescence of fibroblasts is induced via miR200c through SASP with the secretion of pro-inflammatory cytokines TGFB2, chemokine CCL5 and growth factor PDGFA, PDGFB. Further, we delineate the mechanisms of reprogramming fibroblasts by miR-200c to recapitulate the pro-tumorigenic effects of CAFs, leading to aggressive cancer with paracrine effects on carcinoma cells with increased mitochondrial activity, reduced apoptosis, excessive proliferation and immunosuppressive features (**Fig. 10I**). Conversely, anti-miR-200c in fibroblasts induces antitumor effects with inhibition of NFκB and HIF activity and invigorating the anti-cancer immune profile.

In this study, fibroblasts with a reduced stemness phenotype via miR-200c drive cancer progression. There is conflicting data regarding the relationship between EMT-MET and stemness [14–16]. Intermediate states of EMT-MET may drive stemness features rather than the two extreme states, which may explain the apparent paradox. Cancer stem cells are a small subpopulation of cells within tumors and hypothesized to be the primary driver of cancer growth and metastasis as well as therapeutic resistance and cancer recurrence. A better understanding of stem cell phenotypes in carcinoma and stromal cells and their effects on cancer aggressiveness are urgently needed. The contribution of miR-200c to cancer aggressiveness may be compartment specific and differ between carcinoma and stromal cells. Future studies will need to determine the precise mechanisms by which miR-200c affects stem cell differentiation while maintaining its classical pathway and senescent features.

Collectively, the trans-differentiated/senescent profile of fibroblasts induced by epigenetic regulation of miR-200c drives a pro-tumorigenic phenotype of CAFs with inflammatory-metabolic reprogramming coupled with activation of heterotypic pathways. Further studies are needed to determine whether this unique CAF phenotype via miR-200c can be targeted therapeutically.

## MATERIALS AND METHODS

### Establishment of cell lines with stable expression of miR-200c and COMMD1-KD

Precursor miR-200c (HmiR0180-MR03-10), anti-miR-200c (HmiR-AN0302-AM04) and control (CmiR0001-MR03, CmiR-AN0001-AM04) lentiviral plasmids were purchased from GeneCopoeia. Four fibroblast cell lines were used to generate stable cell lines overexpressing miR-200c, anti-miR-200c or controls. MEFs and NIH/3T3 fibroblast cells were isolated from C57BL/6 and BALB/C embryos respectively. BJ1 and HS5 human fibroblasts were isolated from foreskin and bone marrow stroma respectively.

Mouse COMMD1 sgRNAs (GUCUAUUGCAUCUGCAGACA, CCCGAAGGCCGUUGUCCCAG and CCAGCUCUAUUAUGGCAACA) were purchased from Synthego. Transfection was performed according to the manufacturer's protocol.

### Cell Culture and treatment

Human MCF7 and MDA-MB-231 breast carcinoma cells, and 4T1 BALB/C breast carcinoma cells as well as human HS5 and NIH 3T3 fibroblasts were purchased from ATCC. AT3 breast carcinoma cells in a C57Bl6/J background were a kind gift from Dr. Scott Abrams at Roswell Park Cancer Center. Human skin fibroblasts immortalized with human telomerase reverse transcriptase catalytic domain but non-transformed hTERT-BJ1 (BJ1) were purchased from Clontech. Cells were grown in DMEM medium supplemented with 10% fetal bovine serum, 100 units/ml penicillin, and 100 units/ml streptomycin. Cells were treated as follows: SAHA (SML0061, Sigma) 1 µM, 5-Aza-2′-deoxycytidine (5-Aza) (A3656, Sigma) 3 µM, H_2_0_2_ (516813, Sigma) 40-100 µM, N-acetyl cysteine (NAC) (A7250, sigma) 10mM, PT2399 (Selleckchem) 3 µM for 24 or 96 hours. Fresh media containing the pharmacological compounds was changed every 24 hours.

### Co-culture system

In co-culture, fibroblasts and carcinoma cells were seeded at a 4:1 ratio in 12-well plate, and the total number of cells per well was 1×10^5^. As controls, homotypic cultures of fibroblasts and carcinoma cells were plated with the same total number of cells as co-culture. To obtain two cell populations, fibroblasts with an RFP/GFP tag were co-cultured with carcinoma cells with a GFP/RFP tag and underwent flow cytometry analysis and cell sorting.

### Antibodies

COMMD1 (NBP2-4633, Novusbio), ZEB1 (NBP2-13159, Novusbio), Sox2 (ab97959, Abcam), KLF4 (ab151733, Abcam), Oct-4 (2750, Cell Signaling), MYC (5605, Cell Signaling), NANOG (D73G4)(4903, Cell Signaling), K8/18 (20R-Cp004, Fitzgerald Industries), P53 (SC-126, Santa Cruz Biotechnology), Phospho-NFκB p65 (Ser536) (93H1)(3033, Cell Signaling), HIF1A(610959, BD), HIF2A(NB100-122, Novusbio), MCT4(sc-50329, scbt), CAV1(610407, BD), B-actin (A5441, Sigma), tubulin (T4026, Sigma), FITC-conjugated anti-CD24, PE-conjugated anti-CD24, APC-conjugated anti-CD44, APC-conjugated anti-PD-L1 (BD Biosciences).

### Proteomic assay

2D DIGE (two-dimensional difference gel electrophoresis) and mass spectrometry protein identification were run by Applied Biomics (www.appliedbiomics.com). Briefly, image scans were performed immediately following the SDS-PAGE with Typhoon TRIO (GE Healthcare) by the protocols provided. The scanned images were then analyzed by Image QuantTL software (GE-Healthcare) and subjected to in gel analysis and cross-gel analysis using DeCyder software version 6.5 (GE-Healthcare). The ratio of protein differential expression was obtained from in gel DeCyder software analysis. The selected spots were picked by Ettan Spot Picker (GE-Healthcare) following the DeCyder software analysis and spot picking design. The selected protein spots were subjected to in-gel trypsin digestion, peptide extraction, desalting and followed by MALDI-TOF/TOF.

### RNA-seq assay

hTERT-BJ1 fibroblasts with miR-200c overexpression and controls were cultured in DMEM medium without FBS and sodium pyruvate for two days. Tumor tissues were collected from mice co-injected with AT3 and MEF WT antimiR-CNL(G1)/antimiR-200c(G2), COMMD1 KD antimiR-CNL(G3)/antimiR-200c(G4). Total RNA was then isolated with Qiagen kit. Whole Transcriptome (total RNA, coding and lincRNA-seq) was performed at Thomas Jefferson Next Generation Sequencing facility.

### Quantitative RT-PCR

Cells were harvested and total RNA was isolated using trizol (invitrogen). TaqMan miRNA assays (Applied Biosystems) were used to quantify the expression of miRNAs according to the manufacturer's instructions. The mean cycle threshold (CT) value was determined from three PCR replicates. Data was reported using the 2^-ΔΔCt^ method. Experiments were normalized to RNU48.

### β galactosidase

The ratio of β-galactosidase in the BJ1 cells was measured using the senescence β-galactosidase kit (cell signaling) according to the manufacturer's protocols. BJ1 fibroblasts overexpressing miR-200c (F200c) or control were cultured in 10% nuserum DMEM media for four days.

### Immunoassays

The levels of cytokines and chemokines in the culture media were assessed using the human inflammation antibody array (RayBiotech) according to the manufacturer's protocols. BJ1 fibroblasts overexpressing miR-200c (F200c) or control were cultured in serum-free DMEM media. Cytokine and chemokine levels in cell-free culture media were measured by antibody arrays at day 2 and 4.

### Luciferase Reporter Assay

NIH3T3 fibroblast cells expressing NFκB or HIF luciferase reporter with miR-200c overexpression or anti-miR-200c and control were seeded in 12-well plates with 1×10^5^ of cells per well. The next day, the media was changed to 10% nuserum in DMEM and cells were incubated under normoxia and hypoxia overnight. Luciferase activity was measured as described ^29^.

### Flow cytometry analysis

CellROX assay: Fibroblasts and cancer cells were seeded under homotypic culture or co-culture for two to four days. Cells were stained with APC-conjugated CellROX and FACS analysis was performed.

Carcinoma cells with an RFP tag were seeded in homotypic culture or co-cultured with fibroblasts for four days. Cells were stained with APC-conjugated anti-PD-L1 antibody.

Click-iT EdU Flow Cytometry Assay kit (C10418, Life Technologies) and FxCycle™ Far Red Stain (F10348, Life Technologies) for cell cycle analysis was performed in standard fashion as described^43^. Apoptosis was quantified using PI or DAPI and annexin-V-APC as described [44].

### Wound healing assay

MCF7 cancer cells growing in 12-well plate as confluent monolayers were mechanically scratched using a 10 µl pipette tip to create the wound. Subsequently, cells were washed with PBS solution twice, and placed in serum-free DMEM media from BJ1 F200c and scramble control cultures. As indicated, wound gaps were microscopically recorded and the wound gap area was compared among groups to measure the cell migratory capacity.

### Lactate assay

Cells were seeded in growth media and the next day changed to media without FBS and sodium pyruvate. Cell culture media was collected on ice and centrifuged at 14000g at 4°C for 5 minutes. Lactate levels were measured with a lactate colorimetric/fluorometric assay kit (Biovision).

### DNA methylation analysis

DNA was isolated from cell lines using FitAmp General Tissue DNA Isolation Kit (P-1003-1, Epigentek). Global DNA methylation 5-mC% change was detected by 5-mC DNA ELISA Kit (D5325, ZYMO Research). CpG islands were predicted using the CpGPlot in EMBOSS from EMBL-EBI. DNA Methylation at premiR-200c promoter region containing CpG rich islands were screened using OneStep qMethyl™kit (D5310, ZYMO Research). Primer sequences were as follows: premiR-200cMe-F2 (CAGGATGGGTAACTGTGTGTG), premiR-200cMe-R2 (CAGAAGTGCCTAGACTGAACTAAC), premiR-200cMe-F3 (AAGTCCCACCTCCTCTAACC) and premiR-200cMe-R3 (GTGTCGCTAGTGTGAAGTTACC).

### PD-L1 IHC assay

Mouse tumor paraffin sections were de-paraffinized and rehydrated. Antigen retrieval was performed in 10 mM citrate buffer pH 6.0 for 10 min with a pressure cooker followed by blocking endogenous peroxidase with 3% H_2_0_2_ for 15 min. Blocking was also performed for endogenous biotin with an avidin-biotin blocking kit. Samples were incubated with 10% goat serum overnight at 4°C. The next day, sections were stained with anti–PD-L1 rabbit primary antibody and incubated with a biotinylated goat-anti-rabbit IgG antibody followed by an avidin-biotinylated horseradish peroxidase complex. Immune-complexes were visualized using the Opti-View DAB IHC Detection Kit followed by an Opti-View Amplification Kit. Immunoreactivity and samples were counterstained with hematoxylin.

### Animal studies

The Institutional Animal Care and Use Committee (IACUC) at Thomas Jefferson University approved all animal protocols (01875, 01607). All experiments were performed in accordance with National Institutes of Health guidelines and the study is reported in accordance with ARRIVE guidelines. Only female mice were used in this study.

To evaluate the tumor-promoting effects of miR-200c in fibroblasts, syngeneic NIH3T3 F200c, anti-miR-200c and control were co-injected with 4T1 cells subcutaneously in the abdominal mammary gland of BALB/c female mice. Carcinoma cells (1 million) and NIH3T3 fibroblasts (0.3 million) were re-suspended in 100 µl of sterile PBS just before injection. After 18 days post-injection, tumors were excised to determine their size. Volumes were calculated using the formula (X^2^Y)/2. P values less than 0.05 were considered significant based on the Mann-Whitney U test. Lungs, livers, spleens and blood were collected to determine metastases and assess clonogenicity quantitatively as described [45]. Tissues were collected from mice, lysed and cells were cultured for selection with 60 µM of 6-thioguanine for 12 days.

Combination treatment with anti–PD-L1 antibody and Paclitaxel in a syngeneic mouse breast cancer model were performed in C57BL/6 female mice. 1.5×10^5^ mouse embryonic fibroblasts (MEFs) overexpressing miR-200c or scramble control were co-injected subcutaneously with 5×10^5^ AT3 cells. Paclitaxel (8 mg/kg) or DMSO was administered intraperitoneally on day 15, 22, 29, and 36 after tumor implantation. Mice were treated intraperitoneally weekly with 200 µg of anti-mouse PD-L1 monoclonal antibody or mouse IgG (clone MOPC 21; BioXcell) on day 18, 25, and 32. Mice were sacrificed, and tumors were excised six weeks post-injection. Lungs and peritoneal implants were collected to determine metastases.

MEFs (1.5×10^5^) WT/COMMD1 KD overexpressing anti-miR-200c or scramble control were co-injected subcutaneously with AT3 cells (5×10^5^). Tumors were excised 24 days post-injection to determine their size.

hTERT-BJ1 cells overexpressing miR-200c, anti-miR-200c and control were co-injected with MCF7 cells into the mammary fat pads of female athymic NCr nude mice (NCRNU; Taconic Farms; at six weeks of age). Carcinoma cells (1×10^6^ cells) and BJ1 fibroblasts (3×10^5^ cells) were suspended in 100 µl of sterile PBS prior to injection. Oophorectomy and estrogen supplementation with 17-estradiol pellet placement (0.72 mg/pellet) were performed two days before injection of the estrogen receptor-positive cell lines MCF7. Tumors were excised four weeks post-injection to determine their size.

### Single-cell RNA-seq analysis

Publicly available scRNA-seq data sets (GEO accession code GSE161529: GSM4909254 and GSM4909296 and E-GEOD-75688) were downloaded. The detailed pre-processing of analysis including QC thresholds, cell annotation and downstream Seurat analysis objects are available from Chen *et al.* [46]. Gene read counts were generated for all samples using Cell Ranger v3.0.2 based on human GRCh38 reference v3.0. Subsequent data analysis was done in R using the Seurat package v4.3 with default parameters, padj<0.05.

Quality control and cell filtering based on (1) the total number of mapped reads for that cell (library size), (2) the number of genes detected and (3) the proportion of reads mapping to the mitochondria. A lower bound of 500 was generally applied to the number of detected genes for each cell type. An upper limit of 0.2 was placed on the mitochondrial proportion with 80–100% of cells below this threshold.

Dead cells were excluded by retaining cells with less than 20% mitochondrial reads, leaving cells for downstream analysis. We performed batch correction with default parameters. Log normalization, variable feature identification (FindVariableFeatures), and z-scoring (ScaleData) were applied to the merged object of all cells, and principal component analysis (RunPCA) with subsequent Uniform Manifold Approximation and Projection (UMAP) dimensionality reduction and graph-based clustering of cells were performed. Markers for each cluster were identified using Seurat's FindAllMarkers command and clusters were assigned to cell populations using published signature genes. Gene expression for genes of interest was then quantified across cell type groupings.

### Statistical analysis

Data was expressed as means ± standard error of the mean. One-way or two-way analysis of variance (ANOVA) was applied to grouped analysis using GraphPad Prism 8 software. The unpaired Mann-Whitney U test was used for statistical comparison of tumor weight and volume in vivo. The R (http://www.R-project.org) was adopted to analyze RNAseq, TCGA breast cancer database data and GEO single cell RNA sequencing data. Statistical significance was defined as p-values of less than 0.05.

## SUPPLEMENTAL MATERIAL

Click here for supplemental data file.

All supplemental data for this article are available online at www.cell-stress.com/researcharticles/2024a-lin-cell-stress/.

## AUTHOR CONTRIBUTION

Z. L. and U. M.-O. were involved in the study design and wrote the manuscript. Z. L., M. R., V.D.B. and M. D.-V. performed the experiments. Z. L., M. R., V.D.B., M. D.-V, D.W-M, M. T., G. U., J.M.C., J.C. and U. M.-O. were involved in data analysis and interpretation.

## Abbreviations:

BRCA: – breast cancer;
CAF: – cancer-associated fibroblast;
EMP: – epithelial-mesenchymal plasticity;
EMT: – epithelial-to-mesenchymal transition;
miR: – microRNA;
MET: – mesenchymal-to-epithelial transition;
ROS: – reactive oxygen species;
SASP: – senescence-associated secretory phenotype;
ScRNASeq: – single cell RNA sequencing;
TME: – tumor microenvironment.
