# Supplementary material for: MiR-200c reprograms fibroblasts to recapitulate the phenotype of CAFs in breast cancer progression
Source: Cell Stress. 2024 Mar 11;8:1–20. doi: 10.15698/cst2024.03.293 (PMC10927306; doi:10.15698/cst2024.03.293)
Supplement: Supplementary file 1 [file ces-08-001-s01.pdf]

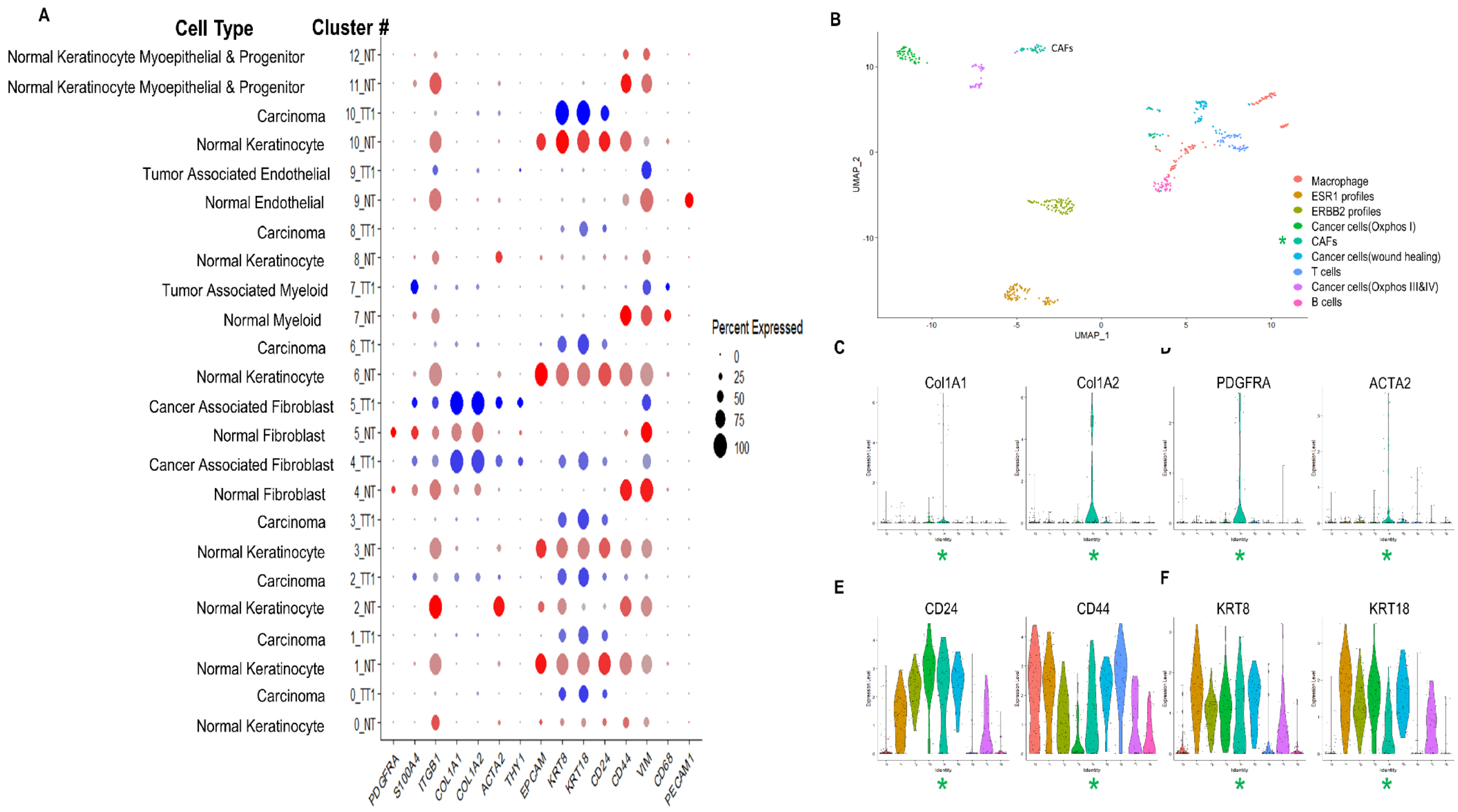

Supplementary Figure S1: Cell type profile and CAFs signature in human breast cancer. (A) DotPlot from scRNAseq showed the expression of specific markers for assigning cell type identity to clusters comprising epithelial cells (ID 0-3, 6, 8, 10), fibroblasts (4, 5), myeloid (7), endothelial cells (9), and myoepithelial & basal progenitor cells (11, 12). **(B-F)** Based on publicly available scRNASeq data of BRCA (E-GEOD-75688). (B)UMAP visualization of assigning cell type feature in breast cancer tissue. * CAFs **(C-F)** Expression of markers in CAFs cluster by VinPlot. * CAFs.

A

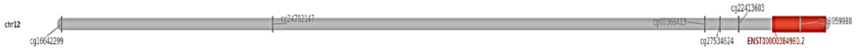

Probe	Chromosome	Start	End
cg27534624	chr12	6963564	6963565
cg24702147	chr12	6962651	6962652
cg22413603	chr12	6963602	6963603
cg18959988	chr12	6963728	6963729
cg16642299	chr12	6962220	6962221
cg00366413	chr12	6963533	6963534

B

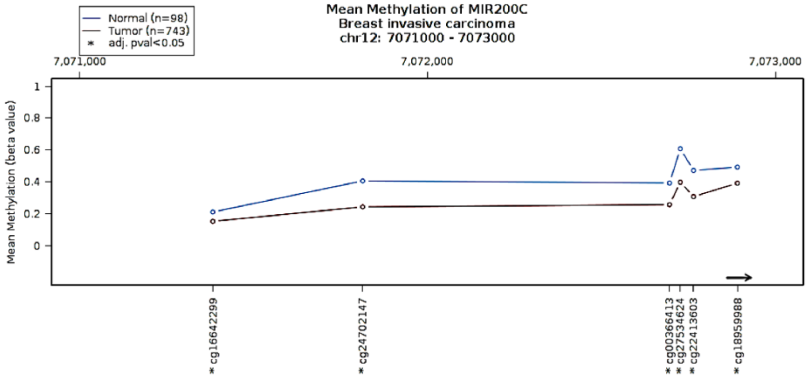

Supplementary Figure S2: Methylation of miR-200c promoter in human breast cancer. (A) Genomic structure of the miR-200c. The plot shows the site of the promoter probes of miR-200c, highlighting transcripts. **(B)** Methylation status of miR-200c promoter region. Six sites of the promoter were hypo-methylated in the tumor versus normal group.

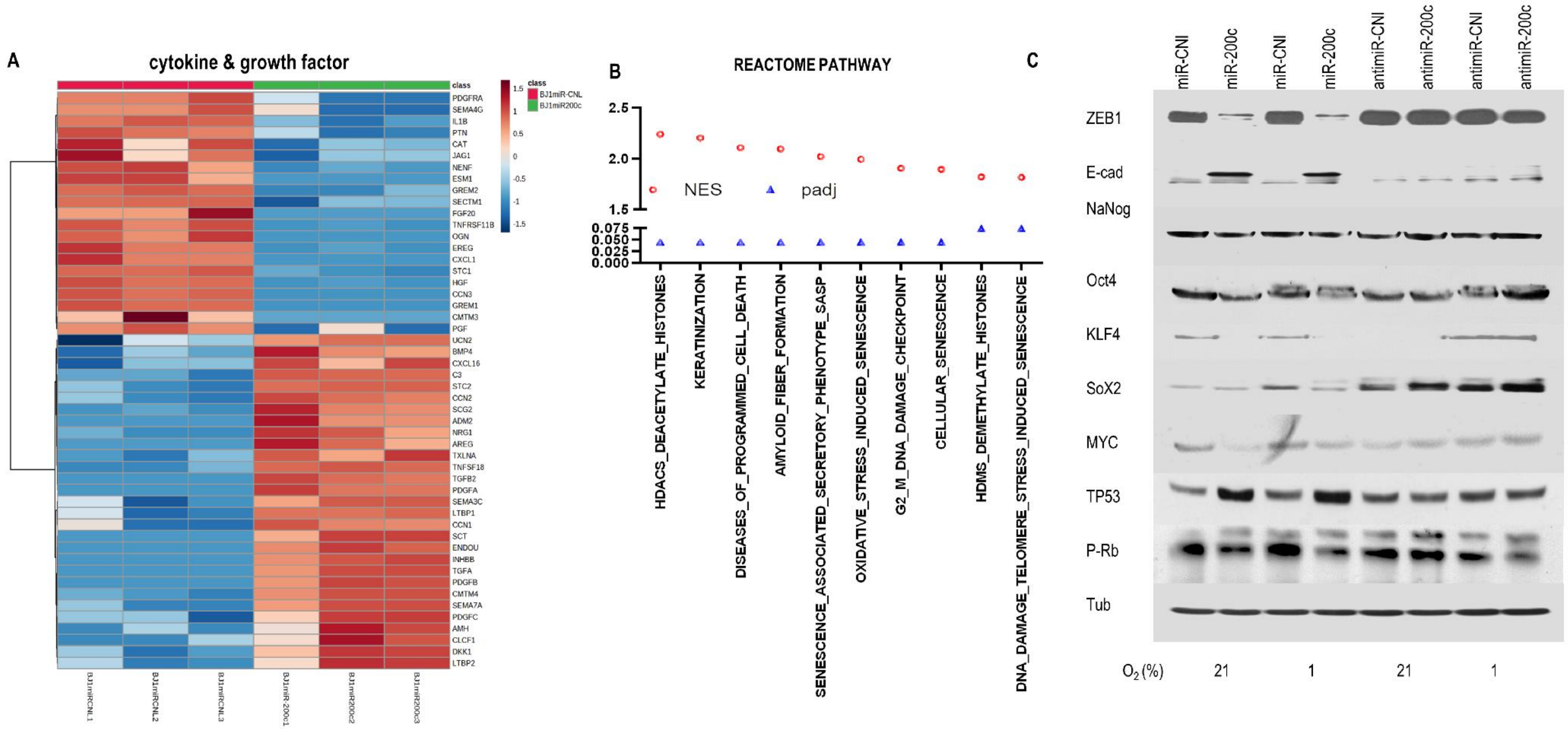

Supplementary Figure S3: Effects of miR-200c on fibroblasts. (A, B) RNAseq data for BJ1miR-200c versus control. **(A)** Heatmap for the expression of cytokine and growth factor. **(B)** GSEA of Reactome pathways driven by miR200c. **(C)** Protein levels of BJ1 cells overexpressing or inhibiting miR-200c and control under normoxic and hypoxic conditions.

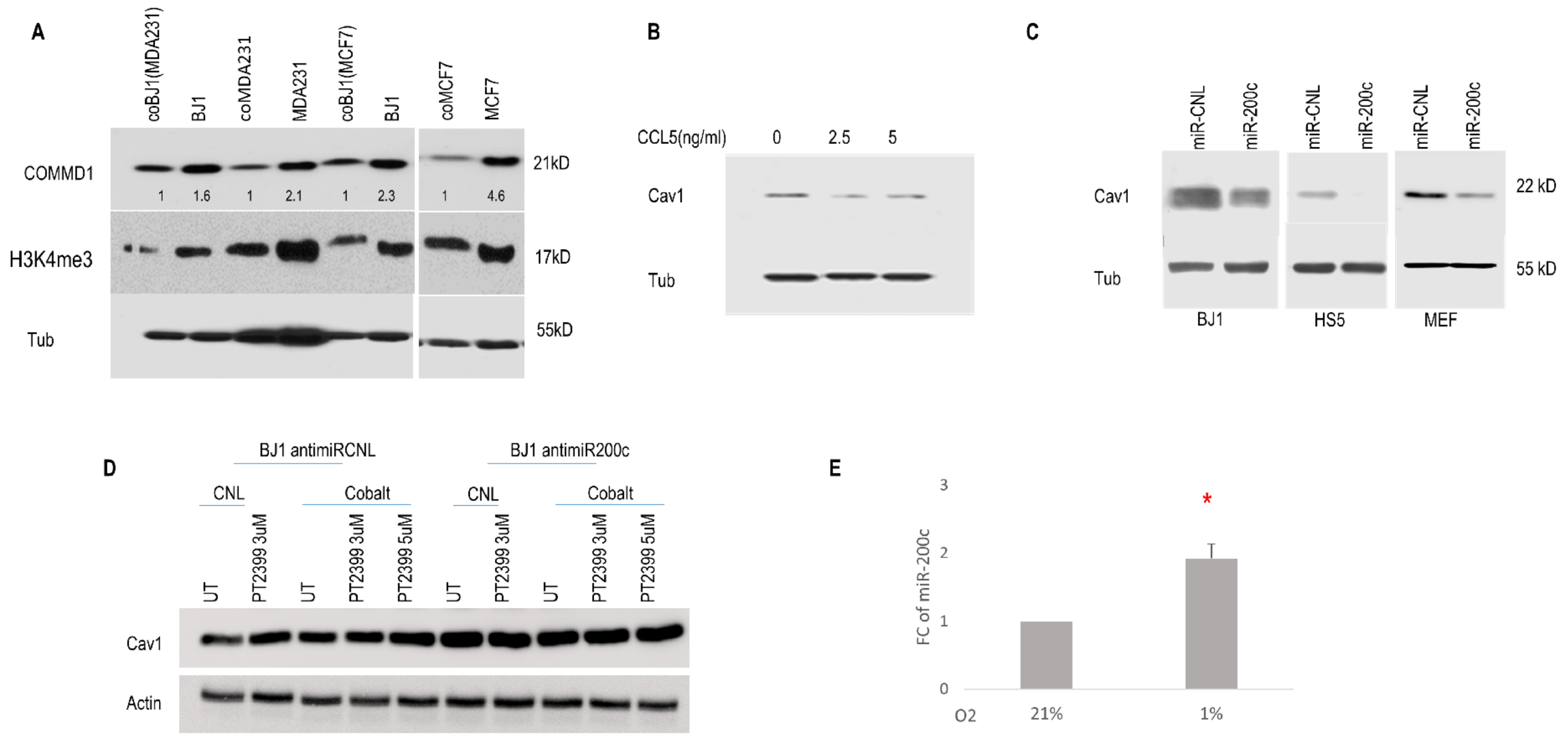

Supplementary Figure S4: COMMD1, CAV1 and miR-200c expression under different conditions. (A) COMMD1 level in fibroblasts and cancer cells during co-culture or homotypic culture for 4 days. **(B, C)** CAV1 expression in BJ1 cells with CCL5 treatment and various fibroblasts with miR-200c overexpression or control. **(D)** CAV1 expression in BJ1 cells expressing anti-miR200c and control with 3uM of HIF antagonist PT2399 and 200uM of cobalt treatment for 48hr. **(E)** Fold change of miR-200c expression in BJ1 fibroblast cells under normoxic and hypoxic condition.

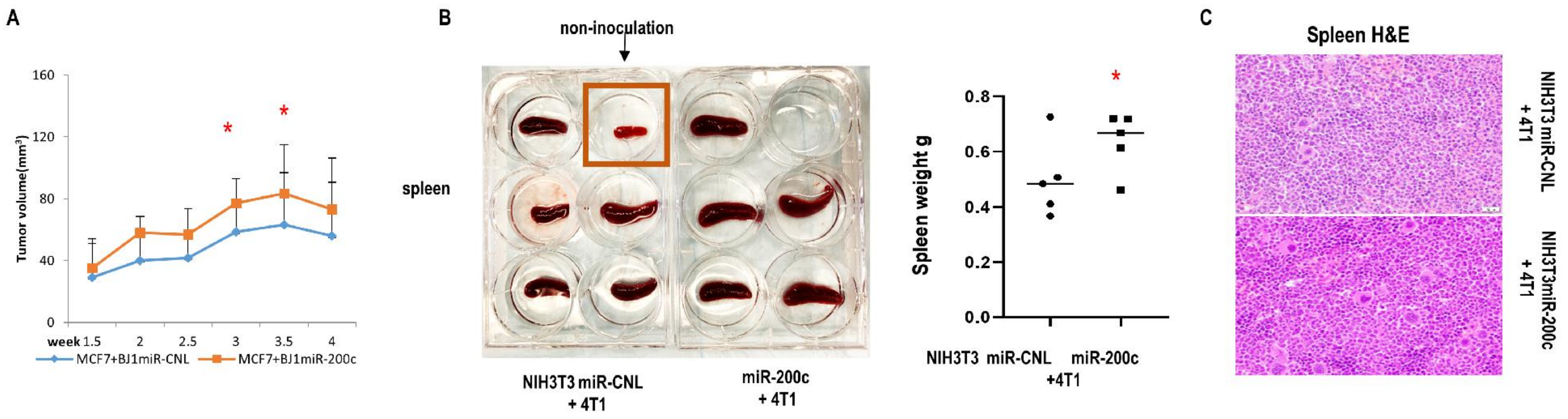

Supplementary Figure S5: Effect of miR-200c in fibroblasts on tumor growth and spleen inflammatory feature. (A) Tumor growth. MCF7 cells co-injected with BJ1 expressing precursor miR-200c and scramble control. Tumor volume was measured at chasing time and after resection at 4 weeks post-injection. (n=8-10, asterisk indicates P<0.05). Error bars denote ±SD. **(B, C)** Co-injection of 4T1 cells and miR-200c NIH3T3 fibroblasts or controls. **(B)** Spleen weight and representative images are shown. The top small box highlights the spleen from a non-inoculated mouse. **(C)** Representative H&E staining of spleens (scale bar: 20 µm).

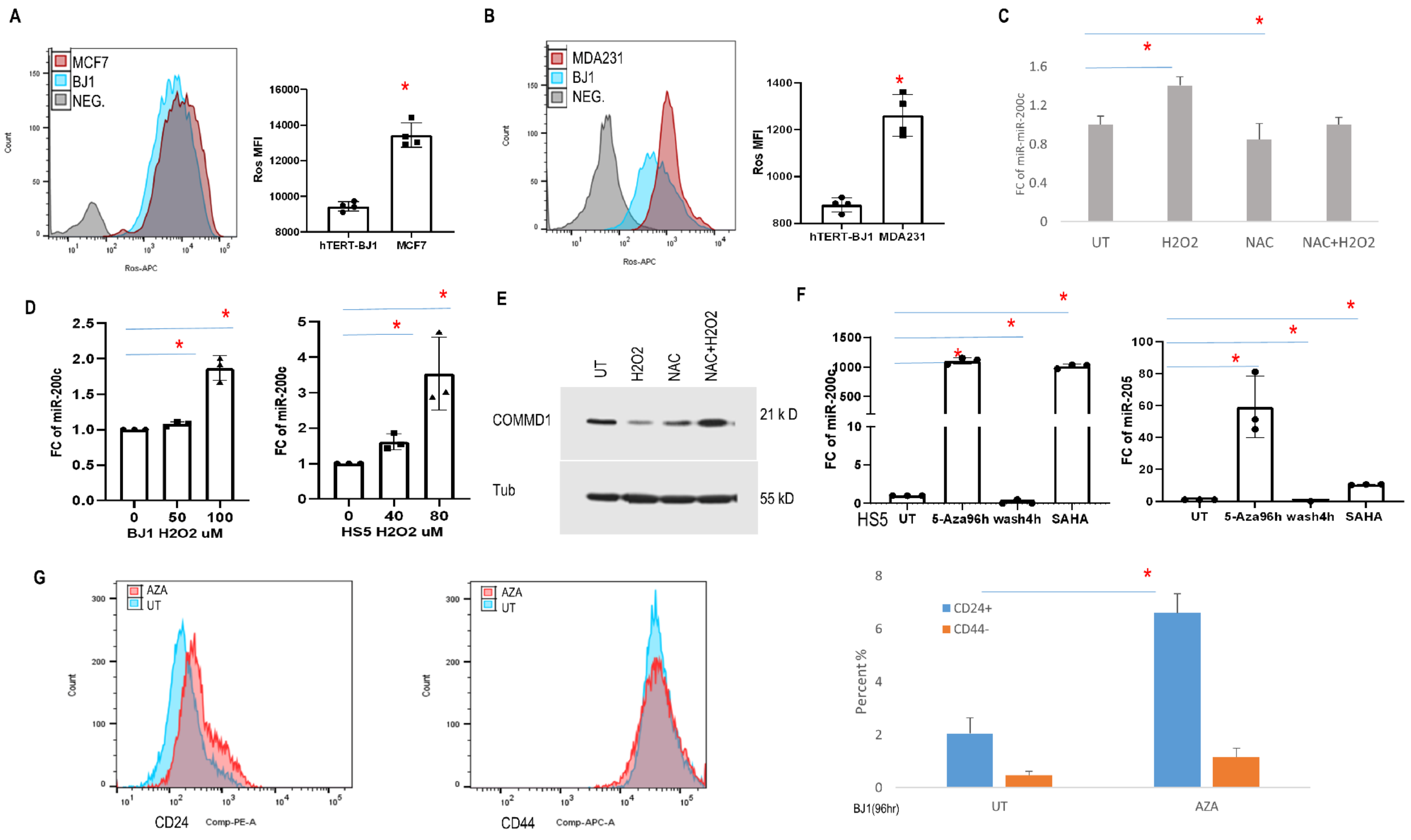

Supplementary Figure S6: Multiple of mechanisms regulated miR-200c and miR205 expression of fibroblasts. (A) Flow chart showed ROS level in BJ1 fibroblasts versus cancer cells MCF7. **(B)** Flow chart showed ROS level in BJ1 fibroblasts versus cancer cells MDA231. **(C)** Fold change of miR-200c in BJ1 cells exposed to H_2O_2, NAC for 16 hours and 2 hours NAC pretreatment with H_2O_2. **(D)** Fold change of miR-200c in fibroblasts exposed to H_2O_2. **(E)** Effect of H_2O_2 on COMMD1 expression in BJ1 cells for 16 hours and 2 hours NAC pretreatment with H_2O_2. **(F)** Fold change of miR-200c and miR-205 in HS5 fibroblasts exposed to drugs regulating the epigenetic repressive state. *p<0.05. **(G)** Gain of CD24 and loss of CD44 ratio following 3um of Aza treatment for 4 days with fresh media daily in hTERT-BJ1 cells.
